# Body composition phase angle: A potential predictor of vitamin D status in early pregnancy

**DOI:** 10.1002/fsn3.3722

**Published:** 2023-10-12

**Authors:** Ziqin Wang, Huiqun Wang, Dan Zheng, Jing Liu, Yanping Liu

**Affiliations:** ^1^ School of Public Health, the Key Laboratory of Environmental Pollution Monitoring and Disease Control, Ministry of Education Guizhou Medical University Guiyang China; ^2^ Department of Maternity Health Guiyang Maternal and Child Health Care Hospital Guiyang China; ^3^ Department of Obstetrics Guiqian International General Hospital Guiyang China; ^4^ Department of Clinical Nutrition Peking Union Medical College Hospital Beijing China

**Keywords:** body composition, early pregnancy, nutritional status, phase angle, vitamin D

## Abstract

Phase angle and vitamin D can reflect the state of the body cells, and the two may interact with each other. Therefore, this study was conducted to find out the relationship between PA and vitamin D. Taking women in early pregnancy as our study subjects, we found that PA had a positive effect on vitamin D levels. Body composition phase angle, as a noninvasive, easy‐to‐operate, and easy‐to‐monitor indicator, can be used an early screening index for vitamin D nutrition levels in early pregnancy, and the cutoff value was 4.95°.

## Graphical abstract

The nutritional status of vitamin D was positively correlated with phase angle, when the vitamin D level was less than 30ng/mL, the phase angle also decreased. It suggests that body composition phase angle, as a safe and noninvasive indicator, can be used as an early screening index for vitamin D nutrition levels in early pregnancy.

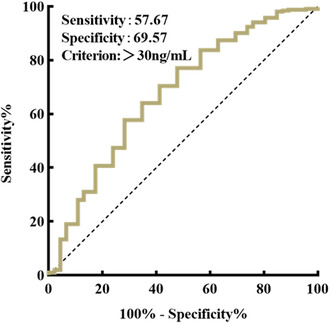

## 1 INTRODUCTION

Body composition measurement is a commonly used method of assessing nutritional status, reflecting the proportion of fat, protein, and other major chemical components of the body, which provides insight into the nutritional status of the body and helps to understand the possible mechanisms of health and disease (Kuriyan, 2018). Bioelectrical impedance analysis (BIA) is a simple, noninvasive, and safe method for measuring body composition (Saqlain et al., 2019); the phase angle (PA) in the BIA measurement result can be calculated from the inverse tangent function of the reactance–resistance ratio. The “conductive part” of the body includes the extracellular fluid, intracellular fluid, and other conductive components at the cellular level, which generate the impedance to the current. The inside and outside of the cell membrane has a voltage difference equal to the capacitance, the PA is related to the integrity and continuity of the cell membrane, and it can represent the health status of the cells in the body, it is generally believed that the PA in ranges between 5° and 7° indicates good cellular function and nutritional status (Basile et al., 2014; Genton et al., 2020; Gonzalez et al., 2016; Sergi et al., 2017). Although the biological significance of PA is not fully understood, it has been widely used as an important prognostic indicator in a variety of clinical situations. Some studies have used PA as a prognostic indicator in cancer, AIDS, dialysis, and other clinical situations, showing that the smaller the PA value, the worse the prognosis or shorter the survival time (Norman et al., 2012).

Recent studies have shown that vitamin D is not only closely related to calcium absorption, but also plays a role in many physiological processes in the body (Wilson et al., 2017). The body obtains vitamin D through skin synthesis by UV exposure and food intake, the former is the main source of vitamin D. About 80% of vitamin D is formed from 7‐dehydrocholesterol in subcutaneous tissues after UV irradiation (Holick, 2007; Jean et al., 2017; Trump & Aragon‐Ching, 2018). Because dietary sources of vitamin D are extremely limited and its subcutaneous synthesis is closely linked to climatic conditions such as sunlight, the risk of vitamin D deficiency is high and vitamin D deficiency has become a global public health problem (Sizar et al., 2022).

Studies show that vitamin D nutrition status during pregnancy are strongly associated with adverse pregnancy outcomes such as preeclampsia, gestational diabetes, and preterm birth (Palaniswamy et al., 2015). The prevalence of vitamin D deficiency among pregnant women is 40% due to the increased nutritional needs of the mother and fetus during pregnancy and the reduced time spent outdoors (Wheeler et al., 2018). Vitamin D has a role in maintaining blood calcium stability, and calcium homeostasis is negatively correlated with blood pressure, plus vitamin D regulates the renin–angiotensin system; therefore, vitamin D deficiency can cause preeclampsia (Cardús et al., 2006). Vitamin D affects insulin secretion and sensitivity, maintains glucose tolerance, enhances calcium absorption in the duodenum and kidneys, and thus activates intracellular signaling in the insulin cells, thereby affecting glucose homeostasis, so when vitamin D is insufficient or deficient, the risk of developing diabetes during pregnancy increased (Hu et al., 2018; Xuan et al., 2013). Vitamin D initiates the function of Toll‐like receptors of the innate immune response by acting on inflammatory and immunomodulatory processes, and due to impaired induction of antimicrobial peptides mediated by Toll‐like receptor function in macrophages, vitamin D‐deficient individuals are prone to preterm birth (Bodnar et al., 2013; Qin et al., 2016). Therefore, monitoring of vitamin D nutritional status during pregnancy is not only closely related to maternal health, but also affects fetal development, it is an important part of nutritional management during pregnancy. Vitamin D synthesized from subcutaneous endogenous sources and obtained through diet first needs to be catalyzed by D_3_‐25‐hydroxylase in the liver to produce 25‐(OH)‐D_3_, and then converted to 1,25‐(OH)_2_‐D_3_ by 25‐(OH)‐D_3_‐1α‐hydroxylase in the kidney before it becomes physiologically active. Serum 25‐(OH)‐D_3_ is currently recognized as a reliable indicator for evaluating serum vitamin D levels in humans due to its good stability and long half‐life in the blood (Nakamura et al., 2019). Serum 25‐(OH)‐D_3_ can assess the vitamin D nutritional status of the body, but this method is invasive and expensive, making it difficult to monitor the vitamin D nutritional level during pregnancy, so it is important to choose a noninvasive and sensitive vitamin D nutritional screening indicator for nutritional management during pregnancy.

There are many factors that affect the vitamin D status of body, such as age, gender, and chronic disease (Mwango et al., 2021), previous studies have used BMI as an indicator to describe the vitamin D nutritional status of the adult, and usually serum vitamin D is lower in obese people (Walsh et al., 2017), however, BMI does not distinguish between lean body mass, fat content, and the proportion of whole‐body water. In addition, the subjects of this study are pregnant women, whose body composition changes at this stage compared with normal adults, so how to find stable, safe, and noninvasive indicators to predict the nutritional status of vitamin D during pregnancy is an urgent problem we need to solve. Nutrition in early pregnancy not only affects the health of the mother, but also affects the growth and development of the fetus. Since early pregnancy is not yet fully adapted to the role, it is often easy to ignore nutritional problems, so it is important to study the nutritional intake of women in early pregnancy. The PA reflects the state of body cells, the larger the PA, the better the cell structure and function. Vitamin D has a regulatory effect on cell proliferation and differentiation of many tissues through activation of receptors in the nucleus, and its nutritional status is closely related to cell structure and function. When the vitamin D status of body changes, the cell structure and function are affected, and the PA, which reflects the cell state, changes accordingly. Therefore, taking women in early pregnancy as the study subjects, this study aims to understand the vitamin D nutritional status and body composition indices, to investigate the predictive role of PA on vitamin D, and to analyze the relationship between the two, so as to provide reference for better health care during pregnancy.

## 2 SUBJECTS AND METHODS

This study was conducted according to the guidelines laid down in the Declaration of Helsinki and all procedures involving human subjects were approved by the ethics committee of Peking Union Medical College Hospital (Protocol ID: ZS‐1997). A total of 323 pregnant women in early pregnancy who underwent maternity examinations at Guiyang Maternal and Child Health Care Hospital from November 2021 to July 2022 were included in the cross‐sectional study. Inclusion criteria were: (1) permanent residents (≥6 months of residence) who were registered at the hospital; (2) <14 weeks of gestation; (3) voluntarily participated in this study and signed an informed consent form. Exclusion criteria included: (1) those with severe liver, kidney, heart, and other vital organs and endocrine system diseases; (2) those who are taking drugs that affect serum vitamin D metabolism (e.g., phenobarbital).

### 2.1 General information collection

Through face‐to‐face interviews, questionnaires were used to collect basic information such as age, education level, income level, ethnicity, and number of pregnancies and births of pregnant women.

### 2.2 Serum vitamin D measurement

About 5 mL of blood sample was taken from each subject after overnight fasting, and serum vitamin D levels were determined using high‐performance liquid chromatography mass spectrometry analysis. The testing instrument was liquid chromatography tandem mass spectrometer (API4000) from AB, the reagents were standards and internal standards from Sigma, and quality control products from RECIPE, Germany, and other instruments included Thermo Sorvall ST16R high‐speed cryo‐centrifuge and Anjel 96‐well nitrogen blowing instrument from the United States. Serum (200 μL) was taken and added into acetonitrile solution for protein precipitation, and liquid–liquid extraction was carried out using methyl *tert*‐butyl ether, the supernatant was taken and diluted with acetonitrile, blown dry by nitrogen, and fixed at 100 μL for the detection of vitamin D level. According to the serum vitamin D level, the body's vitamin D nutritional status can be classified as normal or low, and the criteria are: normal vitamin D (25‐(OH)‐D_3_ ≥30 ng/mL), low vitamin D (25‐(OH)‐D_3_ <30 ng/mL) (Holick et al., 2011).

### 2.3 Body composition measurement

The Korean InBody 770 body composition analyzer was used to obtain body composition indices such as height, weight, and PA of women in early pregnancy. The body mass index (BMI) was calculated based on height and weight, and the formula is: BMI = weight (kg)/height (m)^2^, the classification criteria are: thin (BMI <18.5 kg/m^2^), normal (18.5 ≤ BMI < 24.0 kg/m^2^), overweight or obese (BMI ≥24.0 kg/m^2^) (Zhou & Cooperative Meta‐Analysis Group of the Working Group on Obesity in C, 2002). The specific measurement conditions were as follows: the metal accessories were removed from the subject and the urine and stool were emptied before the measurement. All subjects were measured while standing with their feet on the test bench, heels placed on the foot electrodes, both hands holding the handles, body upright, and both arms naturally relaxed and hanging down at the sides of the body.

### 2.4 Statistical analysis

Data were organized and logically checked for errors using Excel 2019, and all statistical analyses of data were performed with SPSS statistical analysis software (version22.0; SPSS, Inc.). Measurement data (e.g., serum vitamin D, height, PA) were expressed as mean and standard deviation ($\bar{x}$±s), and the analysis of vitamin D status under different BMI categories was performed using one‐way ANOVA method, and further two‐by‐two comparisons were made using the LSD method; two independent samples *t* test was used to analyze the difference between body composition indices under different vitamin D levels. Enumeration data (e.g., age, education level, income level) were expressed as composition ratio (*n* [%]), and χ^2^ test was used for comparison between groups. Correlation analysis was performed using Pearson's correlation with stepwise multiple linear regression using serum vitamin D level as the dependent variable. The sensitivity and specificity, the area under the cure (AUC), and the cutoff value of the PA for detecting serum vitamin D levels were determined by the receiver operating characteristic curve (ROC curve). All *p* values reported were two tailed and the level of statistical significance was set at *p* < 0.05.

## 3 RESULTS

### 3.1 General information

Table 1 shows the general information of the participants. A total of 323 women in early pregnancy were included in this study. Their serum vitamin D level was 16.91 ± 7.30 ng/mL and 300 (92.88%) had low vitamin D nutritional status. Serum vitamin D levels in early pregnancy were correlated with age and number of pregnancies, with statistically significant differences between the normal vitamin D and low vitamin D groups.

**TABLE 1:** General information of women in early pregnancy.

	Normal vitamin D	Low vitamin D	*χ* ^2^	*p*
Age (≥35 years), *n* (%)				
Yes	9 (2.79)	43 (13.31)	9.725	.002
No	14 (4.33)	257 (79.57)		
Education level, *n* (%)				
High school and below	8 (2.48)	72 (22.29)	1.333	.248
Above high school	15 (4.64)	228 (70.59)		
Income level, *n* (%)				
<5000 yuan RMB	10 (3.10)	101 (31.27)	0.967	.617
5000–10,000 yuan RMB	9 (2.79)	131 (40.56)		
≥10,000 yuan RMB	4 (1.24)	68 (21.05)		
Han nationality, *n* (%)				
Yes	17 (5.26)	212 (65.63)	0.109	.741
No	6 (1.86)	88 (27.24)		
Primigravida, *n* (%)				
Yes	10 (3.10)	188 (58.20)	3.316	.069
No	13 (4.02)	112 (34.67)		
First pregnancy, *n* (%)				
Yes	4 (1.24)	122 (37.77)	4.864	.027
No	19 (5.88)	178 (55.11)		

### 3.2 Vitamin D status at different BMI levels

**FIGURE 1:**
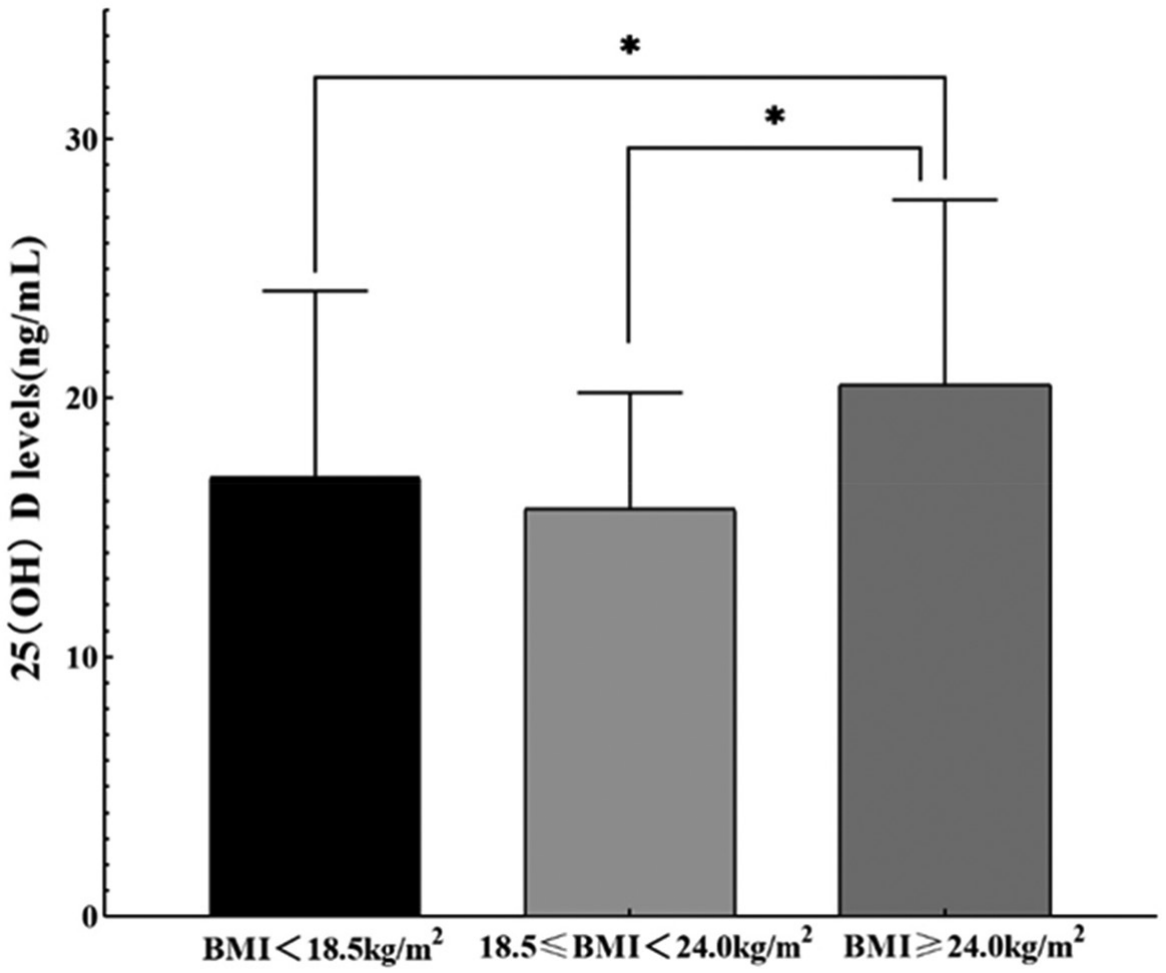
Vitamin D levels under different BMI classifications. *Indicates *p* < .05.

Figure 1 shows the vitamin D levels under BMI classifications. Using one‐way ANOVA method, the results showed statistically significant differences in serum vitamin D levels at different BMI levels. Two‐by‐two comparison using the LSD method showed statistically significant differences between groups in the BMI overweight/obese group and the BMI lean and BMI normal groups, respectively.

### 3.3 Body composition status at different vitamin D levels

Table 2 describes body composition parameters of women in early pregnancy at different vitamin D levels. The differences in weight, BMI, body fat, and PA in women in early pregnancy were statistically significant at different vitamin D levels.

**TABLE 2:** Body composition at various vitamin D levels.

Parameters	Normal vitamin D	Low vitamin D	*t*	*p*
Height ($\bar{x}$ ± s, cm)	157.4 ± 6.1	159.0 ± 5.1	−1.312	.190
Body weight ($\bar{x}$ ± s, kg)	58.0 ± 8.9	54.1 ± 7.5	2.322	.021
BMI ($\bar{x}$ ± s, kg/m^2^)	23.4 ± 3.1	21.4 ± 2.7	2.922	.007
Body fat ($\bar{x}$ ± s, kg)	19.4 ± 5.4	16.9 ± 4.6	2.438	.015
Fat free mass ($\bar{x}$ ± s, kg)	38.6 ± 4.5	37.8 ± 4.1	0.929	.354
Skeletal muscle ($\bar{x}$ ± s, kg)	20.8 ± 2.7	20.2 ± 2.5	1.072	.284
Intracellular water ($\bar{x}$ ± s, L)	17.5 ± 2.1	16.8 ± 2.4	1.207	.228
Extracellular water ($\bar{x}$ ± s, L)	10.8 ± 1.2	10.5 ± 1.5	0.945	.345
Muscle mass ($\bar{x}$ ± s, kg)	36.3 ± 4.3	35.5 ± 3.9	0.968	.334
Protein content ($\bar{x}$ ± s, kg)	7.5 ± 0.9	7.4 ± 0.8	0.889	.375
Inorganic salt content ($\bar{x}$ ± s, kg)	2.8 ± 0.3	2.8 ± 0.3	0.543	.588
PA ($\bar{x}$ ± s, °)	5.2 ± 0.6	4.9 ± 0.5	2.979	.003

### 3.4 Correlation analysis of vitamin D and various indicators of body composition

As shown in Table 3, serum vitamin D levels were correlated with all body composition parameters except height and inorganic salt content, and the weak correlation between vitamin D levels and PA remained after controlling for BMI, body fat, and fat free mass (*r* = .113, *p* = .043).

**TABLE 3:** Correlation of vitamin D with body composition parameters.

Parameters	VD levels (ng/mL)	
	*r*	*p*
Height (cm)	.041	.463
Body weight (kg)	.180	.001
BMI (kg/m^2^)	.180	.001
PA (°)	.174	.002
Body fat (kg)	.157	.005
Fat free mass (kg)	.146	.009
Skeletal muscle (kg)	.154	.005
Muscle mass (kg)	.150	.007
Protein content (kg)	.151	.007
Inorganic salt content (kg)	.103	.064
Intracellular water (L)	.131	.019
Extracellular water (L)	.119	.032

### 3.5 Multiple linear regression analysis

As Table 4 shows, stepwise multiple linear regression analysis was performed with serum vitamin D level as the dependent variable and age, BMI, body fat, PA, and body weight as independent variables, in which age and PA had a positive effect on serum vitamin D level.

**TABLE 4:** Multiple linear regression analysis.

Parameters	Multiple linear regression analysis				
	*B*	*SE*	*β*	*t*	*p*
Constant	−4.959	3.921		−1.265	.207
Age	0.450	0.074	.322	6.102	<.001
PA	1.677	0.729	.121	2.299	.022

### 3.6 The ROC curve

**FIGURE 2:**
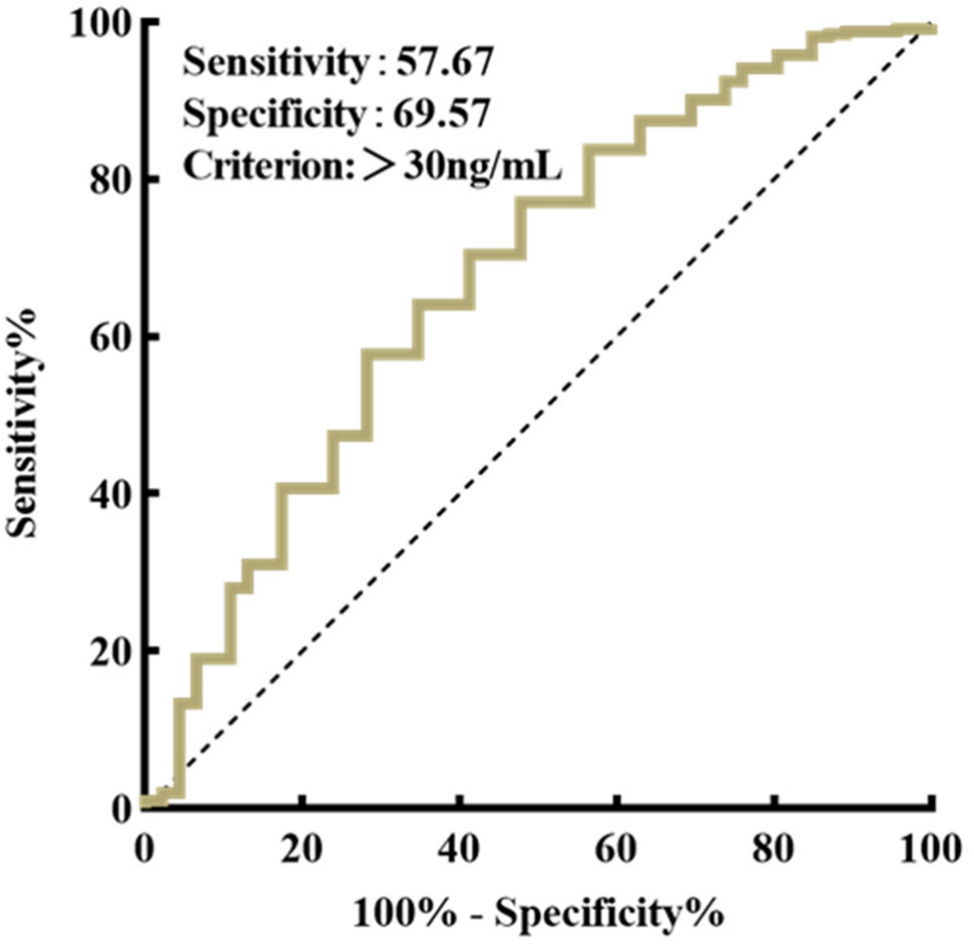
ROC curve for phase angle prediction of vitamin D levels.

As shown in the ROC curve in Figure 2, when vitamin D levels were <30 ng/mL (*p* < .01, AUC = 0.674, *SE* = 0.061, 95% CI 0.554–0.794), the PA decreased and its cutoff value was 4.95°.

## 4 DISCUSSION

### 4.1 Vitamin D nutritional status of women in early pregnancy

The results of this study showed that 92.88% of the subjects had low vitamin D nutrition status in early pregnancy, which is higher than the national level reported in our Nutrition and Health Survey (Hu et al., 2021). This result will not only affect the maternal health but also the fetal growth and development, and should be taken seriously. The reasons for this result may be that (1) vitamin D is mainly derived from subcutaneous synthesis, but Guizhou province is located in the inland area of southwest China, bounded by 24°37′ ~ 29°13′ N, it belongs to a warm and humid subtropical monsoon climate with more cloudy and rainy weather (Zhao et al., 2021), and the average annual sunshine time is shorter, in addition, most women use umbrellas when they go out or apply sunscreen, resulting in insufficient subcutaneous synthesis of vitamin D; (2) dietary sources of vitamin D are mainly animal foods such as seawater fish, liver, and cod liver oil preparations (Borel et al., 2015), but due to the dietary habits of the region, the intake of the above foods is low, resulting in low nutritional status of vitamin D.

### 4.2 Factors influencing vitamin D nutritional status in women during early pregnancy

The results of this study showed that compared to overweight or obese people, those with low BMI had worse vitamin D nutrition status. In general, low BMI people have weaker skeletal muscle strength, and the possible mechanism is that vitamin D affects phosphorus uptake by muscles, which affects myocyte energy metabolism and thus muscle function, and vitamin D deficiency is often associated with decreased muscle function (Song et al., 2020). This study showed that the higher the BMI, the better the vitamin D status, probably due to the fact that vitamin D is stored in adipose tissue, and high levels of vitamin D can be found in the adipose tissue of obese people, whose total serum vitamin D stores are higher. In addition, the subjects of this study were women in early pregnancy, and early pregnancy is prone to induce miscarriage because the fertilized egg has not been in the endometrium for long and the union between the embryo and the endometrium is not yet stable (Coomarasamy et al., 2020). Also, in order to preserve the fetus, pregnant women tend to choose to rest indoors, leading to weight gain, and women during pregnancy invariably show a nearly linear weight gain (Zanardo & Giustardi, 2018), and pregnant women with higher body fat content may have a greater capacity for vitamin D storage, which may also contribute to this result. The results of multiple linear regression analysis showed that age had a positive effect on serum vitamin D levels. The older the age, the higher the vitamin D level, which may be due to the fact that older pregnant women have more knowledge and understanding of pregnancy‐related knowledge and pay more attention to vitamin D nutrition, while relatively speaking, younger pregnant women have less experience in pregnancy and knowledge of related nutritional care.

### 4.3 Relationship between vitamin D levels and PA in early pregnancy

We found that vitamin D nutritional status and PA were positively correlated, and the worse the vitamin D nutritional status of the body, the smaller the PA. As shown in the ROC curve, we concluded that when the serum vitamin D level was <30 ng/mL, the PA decreased and its cut‐off value was 4.95°. The functions of vitamin D include maintaining the dynamic balance of calcium and phosphorus in the body and regulating bone metabolism, but most of these functions are achieved by activating receptors in the nucleus of cells, which in turn regulate the transcription levels of vitamin D target genes. When the nutritional status of vitamin D in the body is better, the structure and function of cells are also better. And the PA can reflect the health status of cells in the body, therefore, when the level of vitamin D in the body decreases, the PA will also become smaller. The PA describes the phase difference between the current and voltage generated by the lag of the current penetrating the cell membrane and tissue interface, and is measured directly by the phase‐sensitive device, the PA reflects the distribution of intracellular and extracellular fluid using the geometric relationship between resistance and reactance (Cornejo‐Pareja et al., 2022; Lukaski, 2013; Sardinha, 2018). In physiology, as an indicator of cell membrane integrity and vitality, the PA reflects both the quantity and quality of soft tissues, the larger the PA, the higher the cell density and better the cell quality. It is an indicator of cell health, PA can reflect cell integrity, the larger the PA, the more intact the cell membrane and the better the cell function. Based on this property, PA is used as a screening tool for disease and an indicator for nutritional assessment (Tanaka et al., 2019; Zdolsek et al., 2000). The PA can be used for nutritional assessment of breast cancer patients, with lower PA values in women at higher nutritional risk and greater PA values in patients with good nutritional status as survival time progresses (Morlino et al., 2022). A bioresistance study in cardiac surgery found that PA was a novel biomarker of frailty in cardiac surgery patients and that low PA (<4.5°) was an incremental predictor of mortality, morbidity, and length of stay after major cardiac surgery (Mullie et al., 2018). Because the PA is rapidly and easily available in almost all patients and is closely related to cellular function, it can be a valuable indicator for assessing disease severity. PA is highly sensitive for the evaluation of patients' nutritional status, and changes in PA usually appear slightly earlier compared to general blood biochemical tests.

Vitamin D is closely related to cellular function, and the vitamin D endocrine system is an important component of the interaction between the kidneys, bones, parathyroid glands, and intestines. They maintain calcium levels in the extracellular fluid, a process that is essential for normal cellular physiology. Studies have shown that vitamin D inhibits the proliferation of human leukemia cells and promotes the differentiation of leukemic bone marrow precursors to a more mature and aggressive phenotype (Cao et al., 2017). Vitamin D regulates apoptosis, it protects keratinocytes and primary melanocytes from apoptosis induced by UV radiation or chemotherapy, and in normal tissues, the proapoptotic properties of vitamin D are important for the control of cell proliferation (Dusso et al., 2005). During pregnancy, vitamin D is not only important for the mother, but also has an extraordinarily important role in embryonic development. Vitamin D regulates the contractile proteins of the uterine muscle cells, and when vitamin D is deficient, it reduces the strength of the contractile muscles, leading to prolonged or difficult labor, indicating the need for a cesarean section (Sebastian et al., 2015). It has been shown that vitamin D, by inhibiting the role of nuclear factor‐κB in the anti‐inflammatory pathway, may play a role in reducing the incidence of infection and thus preterm birth (Thota et al., 2014). Vitamin D has an effect on fetal growth and development by regulating calcium homeostasis and parathyroid hormone levels, and vitamin D deficiency increases the risk of low birth weight infants (Agarwal et al., 2018). Vitamin D plays a part in neuronal differentiation, neurotransmission, and synaptic function, and is also involved in the balance of the immune system. When vitamin D is deficient, it may alter T‐cell activation, affecting adaptive immunity and leading to the development of autism (Wang et al., 2016). Despite the close relationship between vitamin D and PA and cellular health, and the critical importance of vitamin D for maternal and infant health, there are few studies on PA and serum vitamin D status during pregnancy. This study conducted a preliminary investigation of the relationship between the two, providing a new perspective for better monitoring the nutritional status of women during pregnancy.

There are some limitations in this study. First, the sensitivity and specificity of this study were 57.67% and 69.57%, respectively, causing such results may be influenced by confounding factors. In the next studies, we will better control the effects of confounding factors, such as refining the experimental design. Second, this study is a cross‐sectional study with a small sample size, and a larger sample size is needed to further clarify whether there is a causal relationship between vitamin D deficiency and PA.

## 5 CONCLUSIONS

In conclusion, low vitamin D nutritional status was prevalent in the surveyed subjects, and the nutritional status of vitamin D was closely related to body composition indicators. PA was associated with the risk of vitamin D deficiency, and vitamin D nutritional status was positively correlated with the value of PA. When the vitamin D level was <30 ng/mL, the PA also decreased, and the cut‐off value of the PA was 4.95°. As a stable, convenient and noninvasive index, body composition PA can be used as a sensitive indicator for early detection of vitamin D deficiency. However, because this study is a cross‐sectional study, it is not yet possible to infer whether there is a causal relationship between serum vitamin D deficiency and PA, and a larger sample size study is needed to verify the role of PA in the health screening of pregnant women and to better assess their nutritional status.

## AUTHOR CONTRIBUTIONS

**Ziqin Wang:** Conceptualization (equal); data curation (lead); formal analysis (lead); investigation (equal); software (equal); validation (equal); visualization (lead); writing – original draft (lead); writing – review and editing (lead). **Huiqun Wang:** Conceptualization (equal); formal analysis (supporting); funding acquisition (lead); methodology (equal); project administration (equal); resources (equal); software (equal); supervision (equal); validation (equal); writing – review and editing (equal). **Dan Zheng:** Investigation (equal); project administration (equal); resources (equal); software (equal); supervision (equal). **Jing Liu:** Investigation (equal); methodology (equal); resources (equal). **Yan Ping Liu:** Project administration (equal); supervision (equal).

## FUNDING INFORMATION

This work was supported by the first‐class discipline construction project in Guizhou Province – Public Health and Preventive Medicine (No. 2017[85]).

## CONFLICT OF INTEREST STATEMENT

The authors declare no potential conflicts of interests.

## INFORMED CONSENT STATEMENT

Informed consent was obtained from all subjects involved in the study.

## Data Availability

The data that support the findings of this study are available on request from the corresponding author. The data are not publicly available due to privacy or ethical restrictions.
